# Hospital Preparedness, Resilience, and Psychological Burden Among Clinical Nurses in Addressing the COVID-19 Crisis in Riyadh, Saudi Arabia

**DOI:** 10.3389/fpubh.2020.573932

**Published:** 2021-01-08

**Authors:** Ejercito Mangawa Balay-odao, Nahed Alquwez, Ergie Pepito Inocian, Raid Salman Alotaibi

**Affiliations:** ^1^Nursing Department, College of Applied Medical Sciences, Shaqra University, Shaqra, Saudi Arabia; ^2^School of Nursing, Duquesne University, Pittsburgh, PA, United States; ^3^Dawadmi General Hospital, Al Dawadmi, Saudi Arabia

**Keywords:** clinical nurses, COVID-19 crisis, hospital preparedness, nurses' resilience, psychological burden

## Abstract

In the continuous effort to minimize the devastating effects of coronavirus disease (COVID-19) and to curb the spread of the disease, hospital preparedness and resilience play significant roles in the psychological well-being of clinical nurses given that their work demands immediate action to adapt and adjust to stressors. Thus, this study investigates the hospital preparedness, psychological burden, and resilience of clinical nurses in addressing COVID-19 in Riyadh, Saudi Arabia. A total of 281 clinical nurses participated in the survey from April 2020 to June 2020. Results show that clinical nurses perceived a high self-assessed COVID-19 hospital preparedness (49.65, *SD* = 2.30); high self-assessed nurses' resilience (4.03, *SD* = 0.36); and most have normal levels of depression, anxiety, and stress. The variables were predicted to be statistically significant (*F*_18,262_ = 4.14, *p* = 0.001) and accounted for 16.8% of the variance in the nurses' perception of hospital preparedness (*R*^2^ = 0.221; adjusted *R*^2^ = 0.168). The regression analysis was statistically significant (*F*_30,250_ = 6.71, *p* = 0.001) and accounted for 38% of the variance in nurses' resilience (*R*^2^ = 0.446, Adjusted *R*^2^ = 0.380). The predictors of depression, anxiety, and stress show that the overall relationship was statistically significant at (*F*_23,257_ = 6.71, *p* < 0.001), (*F*_23,257_ = 6.675, *p* 0.000), and (*F*_23,257_ = 6.692, *p* 0.000) with 31.9% of the variance (*R*^2^ = 0.375, Adjusted *R*^2^ = 0.319), 31.8% of the variance (*R*^2^ = 0.374, Adjusted *R*^2^ = 0.318), and 31.9 % of the variance (*R*^2^ = 0.375, Adjusted *R*^2^ = 0.319), respectively. The findings of this study helps in improving the continuing education program, psychological support, and mental health program to ensure that the needs of clinical nurses are addressed during the outbreak of a disease.

## Introduction

In December 2019, Wuhan, China reported the first case of the coronavirus disease (COVID-19) (1). Saudi Arabi was among the first countries that implemented precautionary measures [e.g., stopping direct flights between Saudi Arabia and China (2); suspension of Umrah pilgrims; and banning of inbound travel from countries with active COVID-19 cases (3)] to prevent the entry of the disease. However, despite the early implementation of the safety measures by the Saudi Arabia government, the first COVID-19 case was reported on March 2, 2020 because of the undeclared travel history of one traveler from Iran (3). The emergence of COVID-19 in Saudi Arabi resulted in the implementation of stricter rules to prevent the spread of the disease. The Saudi government totally suspended Umrah, Mosques were closed, schools and universities shifted to online learning, travel ban was ordered to all affected countries, and quarantine became mandatory for passengers. Individuals who were infected with COVID-19 were offered free hospitalization (4). As of June 7, 2020, Saudi Arabia has an approximate total of 101,914 COVID-19 patients with 712 deaths (5). This number is expected to increase further if the disease remains uncontrolled. The increasing number of COVID-19 cases puts health professionals at risk for the disease. The nursing staff at the frontlines are at most risk of becoming infected with the disease as the human transmission becomes evident with close contact to the carrier source. The unprecedented increase in the number of COVID-19 patients, person under investigation (PUI), and person under monitoring (PUM) necessitates a response from hospital management through the implementation of guidelines and procedures within a facility in coordination with the local authorities.

Hospital preparedness is integral in maintaining health services and controlling the spread of COVID-19. It involves the prevention, containment, management, monitoring, and identification of persons with COVID-19 or those exposed to them by implementing facility protocol (6). These measures include the training of healthcare personnel (HCP) on infection prevention and control policies, rapid identification, and isolation of patients confirmed or suspected to have COVID-19; placement of patients in appropriate isolation rooms; transmission-based precaution with the use of the appropriate personal protective equipment (PPE); moving a patient with confirmed or suspected COVID-19 within the facility; hand hygiene; environment cleaning; and limiting visitor access (7). Chopra et al. (8) projected that the large influx in the cases will continue to challenge bed capacity, equipment, and HCP in hospitals. The American Nurses Association (ANA) (9) emphasized the importance of the proper implementation of protocols and guidelines in managing COVID-19. During the early phase of the COVID-19 pandemic, no hospital has a well-established clinical protocol or treatment; resources, such as PPE, are limited (10); and the work load of nurses increased (11). These factors can result in mental health problems, such as stress, physical and mental fatigue, and irritability, among nurses (10).

Resilience is defined as the ability to face adverse situations while remaining focused and optimistic for the future (9). This attribute is considered vital for clinical nurses who are continually confronted with competing priorities and a complex healthcare system. With the ongoing pandemic and already stretched nursing staffing, clinical nurses are pushed to the edge with even higher work demands. The shortage of PPE increases the threat to their well-being (12). In Wuhan, China, nurses increase the resilience of their colleagues by comforting and helping each other (13). Gratitude from the public also improves nurses' resilience with praiseworthy feedback and positive image (14). At this moment, nurses' resilience is essential for them to respond to this adversity favorably.

The increasing workload and negative effects of a pandemic may give rise to psychological burden among clinical nurses. As a result, their work performance may decline (15). Nurses are working extensively to ensure that the needs of the patients are attended. As nurses help in the journey of addressing this disease, maintaining and improving of their mental health are as important as improving their knowledge and skills on how to defeat the virus. The normal daily activities of nurses are already very stressful (16) and often have a negative effect on their mental health. The COVID-19 outbreak is a stressful situation that causes fear and anxiety among HCP because they have high likelihood of acquiring the disease. HCP in China have been infected with the disease, and many of them are exposed because of the shortage in PPE (13). In a study conducted in Wuhan, China, nurses were found to experience a severe degree of mental health symptoms (17). The unfortunate avoidance of family members and friends due to stigma, lack of PPE, the physical strain of PPE, preparing and keeping up-to-date with the best practices against COVID-19, and the risk of acquiring COVID-19 cause psychological burdens to nurses (18). In the unending continuous to minimize the devastating effects of COVID-19 and to curb the spread of the disease, resilience plays a significant role in the psychological well-being of clinical nurses given that their work demands immediate action to adapt and adjust to stressors. Thus, this study focuses on understanding hospital preparedness, psychological burden, and resilience of clinical nurses in addressing COVID-19.

## Aim of The Study

This study aims to determine the predictors of hospital preparedness in managing COVID-19 patients and the psychological burden and resilience among clinical nurses in addressing the COVID-19 crisis in Saudi Arabia. It also seeks to determine the effects of hospital preparedness in managing COVID-19 patients to nurses' resilience and hospital preparedness in managing COVID-19 patients and nurses' resilience to the psychological burden of clinical nurses in addressing the COVID-19 crisis in Riyadh, Saudi Arabia.

## Method

This cross-sectional design was conducted in two government hospitals in Riyadh Region, Saudi Arabia from April 2020 to June 2020.

### Sampling and Sampling Technique

This study was conducted in two government hospitals in Riyadh, Saudi Arabia. Hospital A is a training hospital, whereas hospital B is a non-training hospital. Both hospitals are COVID-19 designated facilities. These hospitals provide various medical and nursing services for emergency, outpatient, inpatient, and homecare patients. These hospitals also have infectious control and Middle East respiratory syndrome coronavirus (MERS-COV) policies and guidelines, infectious units, and mental health programs for their employees. The study population was composed of Saudi and non-Saudi clinical nurses who met the study inclusion criteria of being employed in the research site hospital. The inclusion criteria for the respondents of the study included nurses who are on active duty during the COVID-19 crisis and license nurse practitioners in Saudi Arabia who are willing to be part of the study. The exclusion criteria were nurses who are on leave during the COVID-19 crisis and those who refused to participate in the study. The study had a total of 281 respondents, and convenient sampling technique was applied. The actual power after *post-hoc* analysis of the G^*^Power program (version 3.1.9.4) using the *t-*test model, two predictor variables, medium expected effect size (*d* = 0.5), and a 5% level of significance is 0.99.

### Data Gathering Instrument

Demographic data were obtained to describe the respondents. Three instruments were used to obtain data: (1) Hospital Preparedness Assessment Tool, (2) the Depression, Anxiety, and Stress Scale−21 (DASS-21), and (3) the Resilience Scale for Nurses. The researcher received approval for the use of DASS-21 and Resilience Scale for nurses.

### Hospital Preparedness Assessment Tool

This hospital preparedness assessment tool was adapted from the Control for Disease Control and Prevention (CDC) protocol. The element of this protocol is “infection prevention and control policies and training for HCP, the process for the rapid identification and isolation of patients with confirmed or suspected COVID-19, patient placement, transmission-based precaution, movement of a confirmed or suspected COVID-19 patient within the facility, hand hygiene, environment cleaning, monitoring, and managing HCP, and visitor access and movement within the facility” (18). The questionnaire includes YES or NO questions, wherein the staff nurses will determine if the item is present or being practiced in their workplace. The scale level content validity index (S-CVI) score averaging method of the tool based on the evaluation of five experts, which include nurse supervisors, nurse educators in the hospital, and a nurse infectious manager, is 1. According to Polit and Beck (19), a S-CVI score of 1 with 3–5 experts is valid.

### DASS-21

DASS-21 is a tool used to measure the psychological burden of nurses in addressing COVID-19. DASS-21 is a 21-item self-administered questionnaire used to assess depression, anxiety, and stress with 7 items. The “depression” part of the DASS-21 measures low mood, motivation, and self-esteem; the “anxiety” part focuses on physiological arousal, perceived panic, and fear; and the stress part involves tension and irritability. Cronbach's α revealed a result of 0.84 to 0.92 for DASS-Anxiety, 0.90–0.95 for DASS-Stress, and 0.96–0.97 for DASS-Depression (20). To determine DASS severity rating, the score of every statement for each scale (e.g., depression, anxiety, and stress) were added. The total score of the items in each group (e.g., depression, anxiety, and stress) were compared with the severity rating of the three scales to determine their severity labels. If the total score of the items in depression subscale is 0–4, 5–6, 7–10, and 14 and above, then it is interpreted as normal, mild, severe, and extremely severe, respectively. Meanwhile, if the total score of the subscale stress is 0–3, 4–5, 6–7, 8–9, and 10 and above, then the severity label is normal, mild, moderate, severe, and extremely severe, respectively. In the stress subscale, a total score of 0–7, 8–9, 10–12, 13–16, and 17 and above is considered normal, mild, moderate, severe, and extremely severe.

### Resilience Scale for Nurses

The Resilience Scale for Nurses is composed of 22 items validated by Ihara et al. (21). The tool has four principal factors that consist of positivity in nursing, which has eight items; interpersonal skills, which has five items; having an anchor in personal life, which has five items; and response to novelty, which has four items. The overall Cronbach's alpha of RSN is 0.84, “positivity in nursing” is 0.87, interpersonal skills is 0.77, “having an anchor in personal life” is 0.76, and a “response to novelty” is 0.63 (22).

### Data Gathering Procedure

A letter of request for permission to conduct a study was forwarded to the research committee of the institution. Upon approval, an abstract was presented for review by the Research Ethics Committee at the Ministry of Health (MOH), Saudi Arabia. Upon ethics approval, a letter was presented to the Nurse and Medical Director of the participating institution. The list of staff nurses was obtained from the head nurse and/or supervisor of the hospital. The researchers/research assistant explained the importance, procedure, and advantages of the study before asking the respondents to sign the consent form. Once permission has been given by the potential respondent, the researcher/research assistant administered the questionnaire to them. The respondent was informed that indicating his/her name is optional, but a tracking number is included to ensure that no respondent was given the questionnaire more than once.

The questionnaires were distributed in the ward assignment of the nurses. The researcher/research assistant distributed three questionnaires to those willing to partake in the study. The respondents were instructed to answer the questionnaire personally and to contact the researcher for any clarifications, concerns, or problems encountered while answering the tool. For busy respondents, the data gathering tool was retrieved after 2 days or depending on the time set by the respondent and researcher. Once the questionnaire has been received by the researcher, it was checked for any missing data. If any item was left without any answer, then the researcher will ask the respondent to answer the missing item(s). The results were analyzed after the data were tallied and tabulated. The collected data were kept in a folder file with a security code in the personal computer of the primary researcher. Only the primary researcher can access the secured folder file.

### Ethical Consideration

The University Research Committee and the MOH of Saudi Arabia ensured the ethical conduct of the study. Confidentiality was maintained by avoiding data that could associate the response of the respondent to their identity. The aims, purpose, and process of research were discussed with the respondents. Respondents were allowed to ask questions and withdraw from the study if they feel that their rights were being violated. Perceived coercion was avoided by ensuring the absence of an existing power relations between the researchers and the respondents or by using a middleman to contact the respondents. The respondent was also informed that no additional data apart from the survey information will be taken.

### Statistical Analysis

Data related to the descriptive data form and the scales used were evaluated in SPSS 23 program version 22.0. Sociodemographic data were presented with mean, percentages, and frequency analysis. The predictors of hospital preparedness, resilience, and psychological burden of nurses, hospital preparedness predicting nurses' resilience, and hospital preparedness and nurses' resilience predicting psychological burden were evaluated using linear multiple regression analysis.

## Results

A total of 281 clinical nurses in Riyadh, Saudi Arabia participated in the study. More than half of the respondents are from hospital A (77.94%). Most of the respondents are females (85.8%), Bachelor of Science in Nursing (BSN) graduates (84.7%), and Christians (75.8%). The mean age was 33.25 (*SD* = 6.38). The largest group of respondents include Filipinos (58.7%) as compared with Saudi national Nurses (12.1%). Most of the respondents are Emergency Room (ER) nurses (31.7%), and more than half have provided care for a confirmed COVID-19 case (65.2%). The mean years of experience of the nurses were 10.66 (*SD* = 6.37). The mean of the self-assessed degree of confidence of nurses on the government response to COVID-19 was 8.07 (*SD* = 1.82), 8.17 (*SD* = 1.47) for public authorities, and 8.34 (*SD* = 1.47), and 8.46 (*SD* = 1.53) for hospital administration and nursing administration, respectively indicating high level of confidence (Table 1).

**Table 1:** Demographics (*n* = 281).

**Variables**	**n**	**%**
**GENDER**		
Female	241	85.8
Male	40	14.2
**EDUCATIONAL ATTAINMENT**		
Diploma	43	15.3
BSN	238	84.7
**NATIONALITY**		
Saudi	34	12.1
Filipino	165	58.7
Indian	82	29.2
**MARITAL STATUS**		
Single	107	38.1
Married	174	61.9
**RELIGION**		
Muslim	43	15.3
Christian	213	75.8
Hindi	25	8.9
**CLINICAL AREA**		
ER	89	31.7
ICU	30	10.7
OPD	25	8.9
Medical-Surgical Ward	82	29.2
Isolation ward	31	11.0
Operating Room	24	8.5
**PROVIDED CARE FOR CONFIRMED COVID-19 CASE**		
No	123	43.8
Yes	158	56.2
**PROVIDED CARE FOR SUSPECTED COVID-19 CASE**		
No	257	91.5
Yes	24	8.5
	Mean	SD
Age	33.25	6.38
Year of experience	10.66	6.37
Degree of confidence on the government	8.07	1.82
Degree of confidence on the public health authorities	8.17	1.47
Degree of confidence on the hospital administration	8.24	1.47
Degree of confidence on the nursing administration	8.46	1.53

### COVID-19 Hospital Preparedness, Resilience, and Psychological Burden of Nurses

The results of the descriptive analyses of the clinical nurses' perception of COVID-19 hospital preparedness, resilience, and psychological burden in Riyadh, Saudi Arabia are presented in Table 2. Most clinical nurses perceived a high self-assessed COVID-19 hospital preparedness, as evidenced by the mean score of 49.65 (*SD* = 2.30). Furthermore, all COVID-19 hospital preparedness subscales were rated as high.

**Table 2:** Results of the descriptive analyses of the different variables (*n* = 281).

**Variable**	**Mean**	**SD**	
COVID-19 Preparedness of the hospital	49.65	2.30	
Infection prevention and control policies and training for healthcare personnel	6.92	0.27	
Process for rapidly identifying and isolating patients with confirmed or suspected COVID-19	11.30	0.95	
Patient placement	9.46	0.83	
Transmission-Based Precautions	3.85	0.53	
Movement of patients with confirmed or suspected COVID-19 within the facility	2.88	0.51	
Hand hygiene	1.95	0.30	
Environmental cleaning	4.66	0.74	
Monitoring and managing healthcare personnel	2.94	0.29	
Visitor access and movement within the facility	5.70	0.70	
Resilience	4.03	0.36	
Positivity in nursing	4.60	0.41	
Interpersonal skill	4.09	0.51	
Having an anchor in personal life	4.05	0.48	
Response to novelty	3.99	0.76	
Depression	9.53	6.49	n (%)
Normal			144 (51.2)
Mild			55 (19.6)
Moderate			66 (23.5)
Severe			16 (5.7)
Anxiety	9.87	6.05	
Normal			102 (36.3)
Mild			19 (6.8)
Moderate			105 (37.4)
Severe			34 (12.1)
Extremely severe			21 (7.5)
Stress	10.12	7.27	
Normal			231 (82.2)
Mild			16 (5.7)
Moderate			24 (8.5)
Severe			8 (2.8)
Extremely severe			2 (0.7)

The clinical nurses perceived a high self-assessed resilience, as shown by the mean of 4.03 (*SD* = 0.36). The subscales of resilience, namely, “Positivity in nursing” (4.60, *SD* = 0.41), “Interpersonal skills” (4.09, *SD* = 0.51), “Having an anchor in personal life” (4.05, *SD* = 0.48), and “Response to novelty” (3.99, *SD* = 0.76), were also perceived as high by the clinical nurses.

The percentage of respondents with a normal level of depression is 51.2% (*n* = 144), 36.3% (*n* = 102) have normal level of anxiety, and 82.2% (*n* = 231) have normal level of stress while addressing COVID-19 crisis. Meanwhile, 23.5 (*n* = 66) of the respondents have moderate depression, 19.5% (*n* = 55) perceived a mild degree, and 5.7% (*n* = 16) had severe depression during the COVID-19 crisis. In terms of anxiety, 37.4% (*n* = 105) have moderate anxiety, 12.1% (*n* = 34) have severe anxiety, 7.5% (*n* = 21) have extremely severe anxiety, and 6.8% (*n* = 19) have mild anxiety during the COVID-19 crisis. Furthermore, during the COVID-19 crisis, 8.5% (*n* = 24) of the respondents have moderate stress, 5.7% (*n* = 16) have mild stress, 2.85% (*n* = 8) have severe stress, and ~0.7% perceived extremely severe stress.

### Multiple Regression Analysis on the Nurses' Perception of Hospital Preparedness

Multiple regression analysis was used to determine the predictors of hospital preparedness in Riyadh, Saudi Arabia during the COVID-19 crisis, as perceived by staff nurses. The variables were predicted to be statistically significant (*F*_18,262_ = 4.14, *p* = 0.001) and accounted for 16.8% of the variance in the nurses' perception of hospital preparedness (*R*^2^ = 0.221; adjusted *R*^2^ = 0.168). As presented in Table 3, hospital, gender, clinical area, provided care with confirmed cases, and degree of confidence on the government effort against the pandemic were revealed as significant factors for predicting the nurses' perception of hospital preparedness. Specifically, nurses who work in Hospital B have a lower mean score of 1.15 (*p* = 0.020, CI = −2.12, −0.19) as compared with those working in Hospital A. A point increase in female respondent mean score indicates a decrease in the mean score of male respondents of 1.32 (*p* = 0.001, CI = −2.08, −0.56). Similarly, medical–surgical (MS) ward nurses and operating room (OR) nurses have a lower mean of 1.30 (*p* = 0.004, CI = −2.19, −0.41) and 2.50 (*p* = < 0.001, CI = −3.75, −1.26) than the mean score of ER nurses, respectively. The nurses who provided care to patients with COVID-19 perceived a higher mean score of 1.22 (*p* = 0.003, CI = 0.41, 2.03) as compared with those who did not provide care to patients with COVID-19. A point increase in the degree of confidence of clinical nurses on the ability of the government to control COVID-19 cases corresponds to a 0.25 (*p* = 0.039, CI = 0.01, 0.49) increase in the perceived hospital preparedness of nurses in Riyadh, Saudi Arabia.

**Table 3:** Result of the multiple regression analysis on the nurses' perception of hospital preparedness (*n* = 281).

**Predictor variables**	**ß**	**SE-*b***	***p***	**95% CI**	
				**Lower**	**Upper**
Hospital	−1.15	0.49	0.020^*^	−2.12	−0.19
Gender	−1.32	0.39	0.001^**^	−2.08	−0.56
Age	−0.10	0.05	0.067	−0.20	0.01
Educational attainment	−0.01	0.56	0.983	−1.11	1.09
Year of experience	0.10	0.06	0.066	−0.01	0.21
**NATIONALITY (REFERENCE: SAUDI)**					
Filipino	−0.78	0.46	0.092	−1.69	0.13
Indian	−0.41	0.54	0.449	−1.46	0.65
**AREA (REFERENCE: ER)**					
ICU	−0.88	0.51	0.087	−1.89	0.13
OPD	0.42	0.56	0.461	−0.69	1.52
Medical-Surgical Ward	−1.30	0.45	0.004^**^	−2.19	−0.41
Isolation Ward	−0.34	0.51	0.507	−1.34	0.66
Operating Room	−2.50	0.63	<0.001^***^	−3.75	−1.26
Provided care with confirmed case	1.22	0.41	0.003^**^	0.41	2.03
Provided care with suspected case	−1.90	0.56	0.001^**^	−3.00	−0.80
Degree of confidence on the government	0.25	0.12	0.039^*^	0.01	0.49
Degree of confidence on the public health authorities	0.04	0.14	0.801	−0.24	0.31
Degree of confidence on the hospital administration	0.04	0.23	0.858	−0.42	0.50
Degree of confidence on the nursing administration	−0.25	0.22	0.250	−0.68	0.18

The dependent variable was the nurses' perception of hospital preparedness. β is the unstandardized coefficients; SE-b is the Standard error.

R^2^ = 0.221, Adjusted R^2^ = 0.168.

* Significant at 0.05,

** Significant at 0.01,

*** Significant at 0.001.

### Multiple Regression Analysis of Nurses' Resilience

Multiple regression analysis was conducted to examine the predictors of clinical nurses' resilience during the COVID-19 crisis. The regression analysis was statistically significant (*F*_30,250_ = 6.71, *p* < 0.001) and accounted for 38% of the variance in nurses' resilience (*R*^2^ = 0.446, Adjusted *R*^2^ = 0.380). As presented in Table 4, age, years of experience, area of assignment of nurses, degree of confidence on the public health authorities and hospital administration, and the hand hygiene subscale of hospital preparedness were revealed as significant factors for predicting nurses' resilience. Specifically, the addition year in the age of the respondent corresponds to 0.02 (*p* = 0.027, CI = 0.00, 0.03) increase in nurses' resilience. The years of experience had a positive influence on nurses' resilience by 0.03 (*p* = 0.001, CI = 0.05, 0.02). The result observed on nurses assigned in the MS and isolation wards had decreased mean scores of 0.16 (*p* = 0.42, CI = −0.25, −0.01) and 0.16 (*p* = 0.020, CI = 0.30, −0.03), respectively, as compared with the mean score of ER nurses. The increase in the degree of confidence of nurses on the government resulted in 0.06 (*p* = 0.001, CI = −0.09, −0.02) decrease in the perceived nurses' resilience. A point increase in the degree of confidence of nurses in hospital administration corresponds to 0.10 (*p* = 0.002, CI = −0.16, −0.04) decrease on nurses' resilience. A point increase in the degree of confidence of nurses on the public health authorities resulted in 0.14 (*p* = < 0.001, CI = 0.10, 0.18) increase in the nurses' resilience. As to the hospital preparedness subscale, a point increase in the mean score of hand hygiene corresponds to 0.31 (*p* = < 0.001, CI = −0.46, −0.15) decrease on nurses' resilience.

**Table 4:** Result of the multiple regression analysis on the nurses' resilience (*n* = 281).

**Predictor variable**	**ß**	**SE-*b***	***p***	**95% CI**	
				**Lower**	**Upper**
Hospital	0.08	0.07	0.281	−0.07	0.22
Gender	0.01	0.06	0.884	−0.10	0.12
Age	0.02	0.01	0.027^*^	0.00	0.03
Educational attainment	−0.04	0.08	0.666	−0.20	0.13
Year of experience	−0.03	0.01	<0.001^***^	−0.05	−0.02
**NATIONALITY (REFERENCE: SAUDI)**					
Filipino	−0.00	0.12	0.996	−0.24	0.24
Indian	0.05	0.14	0.731	−0.22	0.32
Marital status	−0.04	0.06	0.486	−0.15	0.07
Religion (Reference: Muslim)					
Christian	0.06	0.11	0.601	−0.16	0.28
Hindu	0.02	0.13	0.868	−0.23	0.27
**AREA (REFERENCE: ER)**					
ICU	−0.02	0.07	0.832	−0.16	0.13
OPD	0.11	0.08	0.171	−0.05	0.26
Medical–Surgical Ward	−0.13	0.06	0.042^*^	−0.25	−0.01
Isolation Ward	−0.16	0.07	0.020^*^	−0.30	−0.03
Operating Room	0.03	0.18	0.850	−0.32	0.39
Provided care with confirmed case	0.11	0.06	0.064	−0.01	0.22
Provided care with suspected case	−0.01	0.08	0.924	−0.17	0.16
Degree of confidence on the government	−0.06	0.018	0.001^**^	−0.09	−0.02
Degree of confidence on the public health authorities	0.14	0.02	<0.001^***^	0.10	0.18
Degree of confidence on the hospital administration	−0.10	0.03	0.002^**^	−0.16	−0.04
Degree of confidence on the nursing administration	0.00	0.03	0.883	−0.06	0.06
Infection prevention and control policies and training for healthcare personnel	0.03	0.18	0.858	−0.32	0.38
Process for rapidly identifying and isolating patients with confirmed or suspected COVID-19	−0.04	0.02	0.055	−0.08	0.00
Patient placement	−0.00	0.02	0.918	−0.05	0.05
Transmission-Based Precautions	0.08	0.04	0.056	−0.00	0.17
Movement of patients with confirmed or suspected COVID-19 within the facility	0.01	0.05	0.921	−0.09	0.10
Hand hygiene	−0.31	0.08	<0.001^***^	−0.46	−0.15
Environmental cleaning	0.03	0.03	0.411	−0.04	0.09
Monitoring and managing healthcare personnel	0.06	0.12	0.617	−0.17	0.29
Visitor access and movement within the facility	−0.05	0.05	0.288	−0.14	0.04

The dependent variable was the nurses' perception of nurses' resilience. β is the unstandardized coefficients; SE-b is the Standard error.

R^2^ = 0.446, Adjusted R^2^ = 0.380.

* Significant at 0.05,

** Significant at 0.01,

*** Significant at 0.001.

### Predictors of Depression, Anxiety, and Stress

Multiple regression analysis was conducted to examine the predictors of depression of clinical nurses during the COVID-19 crisis in Riyadh, Saudi Arabia. The regression model accounted 31.9% of the variance in depression (*R*^2^ = 0.375, Adjusted *R*^2^ = 0.319), and the overall relationship was statistically significant (*F*_23,257_ = 6.692, *p* < 0.001). As presented in Table 5, gender, educational attainment, nationality, marital status, religion, area of assignment of nurses, degree of confidence on the government, public health authorities and hospital administration, and nurses' resilience were revealed as significant factors for predicting the nurses' psychological burden. Specifically, nurses in Hospital B have a higher depression mean score, by 3.09, than those in Hospital A. Depression score was negatively correlated to age and degree of confidence in public health authorities, which decreased by 0.44 for every increase in age and by 1.17 for every increase in the degree of confidence of nurses to public health authorities. Females have a lower mean score than males by 2.55. Diploma nurses have a lower mean score than BSN by 4.36. Similarly, Filipino and Indian nurses have a lower mean score than Saudi national nurses by 7.10 and 8.97, respectively. Single nurses also have a lower mean score than married nurses by 2.63. Nurses who did not provide care to a COVID-19 patient has a lower mean score by 2.12 than those who provide care to a COVID-19 patient. A point increase in the degree of confidence of nurses on the public health authorities corresponds to a 1.17 decrease on the nurse's depression mean score. Meanwhile, a point of increase in the degree of confidence of nurses on the government and hospital administration resulted in 0.88 and 1.36 increment in the depression mean score of nurses. Isolation ward nurses' depression mean score is higher by 4.35 than ER nurses. A point of increase in the nurses' resilience corresponds to a decrease of 4.09 depression mean score of nurses.

**Table 5:** Predictors of depression, anxiety, and stress (*n* = 281).

**Predictors**	**Depression**					**Anxiety**					**Stress**				
	**ß**	**SE-b**	***p***	**95% CI**		**ß**	**SE-b**	***p***	**95% CI**		**ß**	**SE-b**	***p***	**95% CI**	
				**Lower**	**Upper**				**Lower**	**Upper**				**Lower**	**Upper**
Hospital	3.09	1.34	0.022*	0.46	5.73	−1.27	1.25	0.312	−3.72	1.19	2.56	1.50	0.089	−0.39	5.51
Gender	−2.55	1.03	0.014*	−4.57	−0.53	−2.86	0.96	0.003**	−4.75	−0.97	−2.14	1.15	0.065	−4.40	0.13
Age	−0.44	0.15	0.003**	−0.72	−0.15	−0.10	0.14	0.441	–.37	0.16	−0.29	0.16	0.075	−0.61	0.03
Educational attainment	−4.36	1.50	0.004**	−7.32	−1.41	−5.52	1.40	<0.001***	−8.28	−2.76	−4.44	1.68	0.009**	−7.76	−1.13
Year of experience	0.52	0.16	0.001**	0.22	0.83	0.14	0.14	0.350	−0.15	0.42	0.34	0.17	0.054	−0.01	0.68
**NATIONALITY (REFERENCE: SAUDI)**															
Filipino	−7.10	2.20	0.001**	−11.438	−2.770	−6.17	2.05	0.003**	−10.22	−2.13	−7.14	2.47	0.004**	−12.00	−2.29
Indian	−8.97	2.54	<0.001***	−13.960	−3.972	−8.78	2.37	<0.001***	−13.44	−4.11	−8.82	2.84	0.002**	−14.42	−3.23
Marital status	−2.63	1.03	0.011*	−4.648	–.610	−5.65	0.96	<0.001***	−7.54	−3.77	−4.55	1.15	<0.001***	−6.81	−2.28
**RELIGION (REFERENCE: MUSLIM)**															
Christian	8.96	2.03	<0.001***	4.96	12.96	6.76	1.90	<0.001***	3.02	10.49	10.18	2.28	<0.001***	5.69	14.66
Hindu	6.31	2.34	0.007**	1.71	10.90	6.10	2.18	0.005**	1.81	10.39	8.78	2.62	0.001**	3.63	13.94
**AREA (REFERENCE: ER)**															
ICU	−0.51	1.32	0.703	−3.11	2.10	0.86	1.24	0.489	−1.58	3.29	0.18	1.48	0.903	−2.74	3.10
OPD	−0.44	1.46	0.761	−3.32	2.43	0.06	1.36	0.964	−2.62	2.74	0.95	1.64	0.562	−2.27	4.17
Medical-Surgical Ward	0.89	1.19	0.454	−1.45	3.23	2.20	1.11	0.048*	0.02	4.39	2.55	1.33	0.056	−0.07	5.18
Isolation Ward	4.35	1.33	0.001*	1.74	6.96	2.57	1.24	0.039*	0.13	5.00	4.80	1.49	0.001**	1.87	7.73
Operating Room	1.09	1.69	0.522	−2.25	4.42	4.17	1.58	0.009**	1.05	7.28	5.37	1.90	0.005**	1.64	9.11
Provided care with confirmed case	−2.12	1.09	0.052	−4.27	0.02	−4.76	1.02	<0.001***	−6.76	−2.76	−5.47	1.22	<0.001***	−7.88	−3.07
Provided care with suspected case	0.64	1.48	0.663	−2.26	3.55	1.63	1.38	0.237	−1.08	4.34	1.16	1.65	0.483	−2.09	4.42
Degree of confidence on the government	0.88	0.33	0.008**	0.24	1.53	0.11	0.31	0.711	−0.49	0.72	0.34	0.37	0.350	−0.38	1.07
Degree of confidence on the public health authorities	−1.17	0.40	0.004**	−1.95	−0.38	−0.99	0.37	0.008**	−1.72	−0.26	−1.30	0.45	0.004**	−2.18	−0.43
Degree of confidence on the hospital administration	1.36	0.61	0.026*	0.16	2.57	2.59	0.57	<0.001***	1.47	3.72	2.64	0.68	<0.001***	1.29	3.98
Degree of confidence on the nursing administration	−1.02	0.57	0.073	−2.13	0.09	−1.38	0.53	0.010*	−2.41	−0.34	−1.17	0.63	0.066	−2.41	0.08
Hospital Preparedness	−0.01	0.16	0.947	−0.33	0.31	0.24	0.15	0.108	−0.05	0.54	0.15	0.18	0.391	−0.20	0.51
Resilience	−4.09	1.16	0.001**	−6.37	−1.80	−1.90	1.08	0.081	−4.03	0.23	−1.62	1.30	0.213	−4.18	0.94
R^2^ (Adjusted R^2^)	0.375(0.319)					0.374 (0.318)					0.375 (0.319)				

As to the predictors of the anxiety of clinical nurses during the COVID-19 crisis in Riyadh, Saudi Arabia, the regression analysis was statistically significant (*F*_23,257_ = 6.675, *p* < 0.001) with 31.8% of the variance in anxiety (*R*^2^ = 0.374, Adjusted *R*^2^ = 0.318). Table 5 shows that with other variables held constant, female nurses' anxiety mean scores are lower by 2.86 than those of male nurses. Similarly, nurses with diplomas had a lower mean than BSN nurses by 5.52. Filipino and Indian nurses' anxiety mean scores were lower than Saudi national nurses by 6.17 and 8.78, respectively. MS, isolation ward, and OR nurses have higher anxiety mean scores than ER nurses by 2.20, 2.57, and 4.17, respectively. Nurses who provide care to confirmed COVID-19 patients have lower anxiety mean scores than those who do not provide care to COVID-19 patients by 4.76. A point increase in the degree of confidence of nurses in public health authorities and nursing administration led to a decrease of 0.99 and 1.38 of the anxiety mean score, respectively, whereas a point increase in the degree of confidence of nurses on the hospital administration corresponds to 2.59 increase in the anxiety mean score of nurses.

With regards to the predictors of the stress of clinical nurses during COVID-19 crisis in Riyadh, Saudi Arabia, the regression analysis was statistically significant (*F*_23,257_ = 6.692, *p* < 0.001) with a 31.9% of the variance in stress (*R*^2^ = 0.375, Adjusted *R*^2^ = 0.319). Table 5 shows that with other variables held constant, nurses with a diploma had lower stress mean score than BSN nurses by 4.44. Filipino and Indian nurses' stress mean scores were lower than Saudi national nurses by 7.14 and 8.82, respectively. Single nurses have a lower mean score than married nurses by 4.55. Isolation ward and OR nurses have higher stress mean scores than ER nurses by 4.80 and 5.37, respectively. Nurses who provide care to confirmed COVID-19 patients have lower stress mean scores than those nurses who did not provide care to COVID-19 patients by 5.47. A point increase in the degree of confidence of nurses in public health authorities corresponded to a decrease of 1.30 in anxiety mean score, whereas a point increase in the degree of confidence of nurses on the hospital administration corresponds to a 2.64 increase in the anxiety mean score of nurses.

## Discussion

The finding of this study shows that clinical nurses in Riyadh, Saudi Arabia have high perception of hospital preparedness in combating COVID-19. This finding implies that the hospital is playing its role in preventing and controlling the disease. The hospital is complying with the policies and guidelines set by the WHO and CCDC in response to COVID-19. The guidelines and policies are implemented properly to ensure that staff, patients, and visitors are protected. The finding of the study further shows that the preparedness of the hospital is based on the immediate action executed by the government in response to COVID-19. The government has allocated sufficient funds to ensure hospital readiness in terms of personnel, medicines, availability of equipment, such as hospital beds and ventilators, and other necessary medical supplies, were secured (22). The Saudi government took further extra measures to lessen the consequences of the COVID-19 crisis, which include rapid identification and isolation of confirmed or suspected COVID-19 patients. Currently, no effective medicines or vaccines have been discovered to control and manage COVID-19. For this reason, isolation and identification of symptomatic and asymptomatic persons with COVID-19 can reduce the spread of the virus (23). Thus, early prevention and control are vital in containing COVID-19.

The finding of this study also shows that during this unprecedented crisis, nurses' resilience is very high, thereby showing their ability to adopt in a very stressful situation. The positive characteristics of the respondents that lead to high resilience include age, years of experience, and degree of confidence in public health authorities. As a person ages, their resilience increases, especially in problem-solving and capability to handle emotional problems (24). During the COVID-19 pandemic, as nurses age, their ability to adjust and adapt to the changes in their work became more evident. The years that nurses have spent providing care to infectious and non-infectious patients have contributed to the development of their resilience. This view is supported by Hart et al. (25) who found that competence, flexibility, adaptability, hardiness, and sense of coherence in nurses increase resilience. The actions of the public health authorities by providing guidelines and policies to combat COVID-19 have also helped nurses to fulfill their roles and functions. The policies and guidelines issued by the health authorities help nurses to cope and adjust amid the COVID-19 crisis. These policies and guidelines strengthen the ability of the nurses to protect themselves and deliver services in response to COVID-19 while increasing their competence and confidence in dealing with COVID-19 patients. Being competent and confident are strategies to cope, deal, and handle immediate stressors and enhance resilience (26). The novelty of this disease has also helped nurses to be more engaged in understanding and learning up-to-date information to prevent and control COVID-19. This action is considered a part of the health coping strategies that improve their resilience (27). Nurses use their nursing knowledge and skills to adapt psychologically and prevent psychological burden. This notion is evident in the findings of this study on the increased number of nurses who have normal levels of depression, anxiety, and stress.

Although many studies have proven that most nurses experience psychological burden during a pandemic (17), the findings of our study show otherwise. Most of the nurses in our study did not experience depression, anxiety, and stress. This finding is associated with the positive support received by nurses from the government, society, and their families. The support from their family and the society help nurses increase their sense of pride and professional identity (28). Therefore, the inspiration received by nurses from their support system contributes to their psychological adaptation and adjustment during this pandemic.

However, although most of the nurses in this study have normal psychological status, male Saudi national nurses, as well as Christian, Hindu, and nurses providing direct care to COVID-19, were found to be predictors of nurses' psychological burden. Male nurses have a higher mean score than the female nurses, indicating that the former is more prone to psychological burden during this pandemic than the latter. This finding could be associated with the role of males in the family in Asian countries. Males are usually the breadwinners and provide the needs of their family. The fear of spreading the disease to their loved ones and the financial impact of this pandemic are factors that could increase the psychological burden of male nurses. However, this assumption needs further investigation in future studies. Saudi nurses are prone to psychological burden because of the following reasons: contagion and spreading the virus to their families and friends because they are living with them unlike foreign nurses who live in accommodations provided by the hospital. This finding is consistent with that in China, where Chinese nurses experience fear, depression, anxiety, and stress due to COVID-19 (17).

Christians and Hindu have higher mean depression, anxiety, and stress scores than Islamic nurses because of poor religious coping. According to Carver (29), religion is a coping mechanism used to promote positive well-being. During this pandemic, everyone wants to address their spiritual need by attending church services or consulting with clergies or priests. In Saudi Arabia, the practice of other religious gatherings or services is not allowed, except for Islam. The practice of praying together as a coping mechanism is not allowed. This conflict between religious practices and orientation and culture in Saudi Arabia affects religious coping, thereby increasing their psychological burden. According to Cruz et al. (30), religion is an effective coping mechanism for psychological burden. Religion provides an avenue to cope with a stressful situation and positive support from religious core group members. This view prompted Cruz et al. (31) to posit that the development of a safe working place for Saudi and non-Saudi nurses where they can freely practice their spirituality would be advantageous.

Nurses working in the MS, Isolation, and OR wards have higher psychological burden than ER nurses. MS and OR ward nurses are usually not exposed to COVID-19 patients and have limited clinical experience in providing care to a patient with an infectious disease as compared with isolation ward and ER nurses. Providing care to COVID-19 patients also requires comprehensive and specific management (32). These identified factors increase the psychological burden of MS and OR ward nurses. The experience of an isolation ward nurse has a high mean score in the psychological burden than an ER nurse because of their prolonged exposure to COVID-19 patients. Most patients are admitted at the isolation ward, which predisposes nurses to be more susceptible to contagion. The feeling of being infected increases the psychological burden of nurses (32).

The findings show that nurses' high resilience decreases the mean score of depression and implies that nurses can cope with stressors during this global pandemic and help prevent depression. Self-confidence, calmness, relaxation, and cheerfulness (33) are positive contributors to the emotional stability of nurses. Thus, the finding implies that positive emotions should be strengthened in the psychological dimension to prevent psychological burden.

## Limitation of The Study

The respondents of the study include clinical nurses. Thus, the findings may not be generalized to the other population with different experiences and perceptions in the clinical settings. Nevertheless, it can be replicated. In addition, participants in this study were limited to two settings because of the COVID-19 pandemic. For better representation, future researchers should conduct a wider scale setting. The use of cluster sampling is also recommended to ensure that each facility will have an allocated proportion to generate representativeness of the sample size. The data were gathered using a self-report questionnaire. Thus, the report bias cannot be controlled. There is also limitation in interpreting the psychological burden of the clinical nurses. Since, DASS-21 is not a measure of clinical diagnoses, which means it cannot diagnose depression, anxiety, or stress. Instead, DASS-21 can determine the presence and severity of stress, anxiety, and depression.

## Conclusion and Implications

This research is significant for nursing administrators and staff nurses because it can help them identify the strained aspects of hospital preparedness that need to be improved to strengthen their workplace in the prevention, control, management, and containment of COVID-19. The development of hospital protocols in the handling of confirmed or suspected COVID-19 patients is also effective.

In clinical areas, the findings of this study can help hospital administrators and nursing leaders to identify the strengths and weaknesses of hospital preparedness for confirmed or suspected COVID-19 patients. The hospital can be guided in designing a continuing education program that would enhance hospital preparedness for confirmed or suspected COVID-19 patients.

Nurses have low psychological burden and high resilience. The finding of this study would be of great help in developing psychological support and mental health program to be implemented in times of crisis.

The results of this study may potentially support the scarce data available on hospital preparedness for confirmed or suspected COVID-19 patients, psychological burden, and resilience. The results of this quantitative endeavor can also serve as basis for other similar future research that aim to explore the same topic. This study fills a gap in the literature on hospital preparedness for confirmed or suspected COVID-19 patients, psychological burden, and resilience.

## Data Availability Statement

The raw data supporting the conclusions of this article will be made available by the authors, without undue reservation.

## Ethics Statement

The studies involving human participants were reviewed and approved by Ministry of Health, Saudi Arabia. The patients/participants provided their written informed consent to participate in this study.

## Author Contributions

EB substantially contributed to the conception and design of the work analysis, interpretation of data for the work, drafting the work and revising it critically for important intellectual content, final approval of the version to be published, and agreeing to be accountable for all aspects of the work in ensuring that questions related to the accuracy or integrity of any part of the work are appropriately investigated and resolved. NA, EI, and RA substantially contributed to the conception of the work, the acquisition of data for the work, revising it critically for important intellectual content, final approval of the version to be published, and agreeing to be accountable for all aspects of the work in ensuring that questions related to the accuracy or integrity of any part of the work are appropriately investigated and resolved. All authors contributed to the article and approved the submitted version.

## Conflict of Interest

The authors declare that the research was conducted in the absence of any commercial or financial relationships that could be construed as a potential conflict of interest.
